# Anticancer Potential of Natural Bark Products—A Review

**DOI:** 10.3390/plants10091895

**Published:** 2021-09-13

**Authors:** Ema Burlacu, Corneliu Tanase

**Affiliations:** 1 Residency Department, “George Emil Palade” University of Medicine, Pharmacy, Sciences and Technology of Târgu Mureș, 38 Gheorghe Marinescu Street, 540139 Târgu Mureș, Romania; morariuemma@yahoo.com; 2 Department of Pharmaceutical Botany, “George Emil Palade” University of Medicine, Pharmacy, Sciences and Technology of Târgu Mureș, 38 Gheorghe Marinescu Street, 540139 Târgu Mureș, Romania

**Keywords:** bark extract, metallic nanoparticles, anticancer, antiproliferative, cytotoxic activity

## Abstract

Cell biology, plant-based extracts, structural chemistry, and laboratory in vitro or in vivo experiments are the principal aspects or interfaces that can contribute to discovering new possibilities in cancer therapy and to developing improved chemotherapeutics. Forestry residues can be used for their wealthy resource in polyphenols and other phytoconstituents known for anticancer properties. This review is designed to bring together information on the in vitro or in vivo anticancer potential of woody vascular plants especially the bark extracts (BE) and biosynthesized metallic nanoparticles (BMN) using bark extracts. Type of extracts, main phytoconstituents found in extracts responsible for the anticancer activity, and targeted cancerous cell lines were followed. The literature data were collected via Clarivate Analytics, Science Direct, PubMed, and Google Academic (2011–2021). The search terms were: bark extracts, metallic nanoparticles, silver nanoparticles, gold nanoparticles, anticancer, cytotoxic activity, antiproliferative effect, and antimetastatic potential in vitro and in vivo. All of the search terms listed above were used in different combinations. The literature data highlight the efficaciousness of the BE and BMN as anticancer agents in in vitro experiments and showed the mechanism of action and their advantage of nontoxicity on normal cells. In vitro testing has shown promising results of the BE and BMN effect on different cancer cell lines. In vivo testing is lacking and more data is necessary for drug development on animal models.

## 1. Introduction

Today, cancer, or malignant disease is an alarming situation because of the uncontrolled and abnormal growth of cancer cells. The most undesirable complication of cancer is metastasis and unfortunately this causes death. High mortality in cancer patients is still persistent and the statistics are not very encouraging [1]. Drug therapies are more inefficient due to resistance to chemotherapy and because it destroys the cancer cells along with normal cells [2,3,4]. The ideal anticancer agent should be able to selectively target the cancerous cells and do no harm to the normal cells [5,6].

Cancer is one of the pathological conditions in which apoptosis plays an important role, whether it is abnormal apoptosis or a defective one. That leads to unsuppressed proliferation, which results in cancerous cells that will not naturally die. For that, scientists focused their attention on anticancer strategies for targeting the uncontrollable production of malignant cells and the disorder of apoptosis [7,8]. The collaboration between cell biology, in vitro and in vivo experiments, and structural chemistry will improve and build new anticancer agents [9].

There is a real need to discover new botanical products for developing new anticancer agents. The chemical composition of plants is still poorly studied and this should be the most important source because of their potency in the pharmacological field. Because plants have been traditionally used by humans in healing processes for centuries, and many of them have success that should be an imperative calling for assiduous research in this field. It is necessary to develop new bioactive compounds to meet the need for new drug therapies. For example, Paclitaxel (Taxol) obtained from *Taxus brevifolia* L., and Vincristine, Vinblastine, and Vinorelbine (Vinca alkaloids) extracted from *Catharanthus roseus* G. Don should remind researchers of the endless source of plants as anticancer agents [10].

The current tendency in biotechnology is to recycle and use environmentally friendly products. In this context, forest waste is now widely appreciated as a rich source of natural polyphenols, with therapeutic potential [11]. Polyphenols extracted from plant bark are widely studied, mostly in vitro, and are recommended as antioxidants, antimicrobial agents, and anticarcinogens [10]. Thus, in vitro and in vivo experiments are necessary to validate their use for the production of novel anticancerous drugs with low cost and energy [5,12].

Besides classical plant bark extracts (BE), biosynthesized metallic nanoparticles (BMN) are synthesized using ecofriendly and green-chemistry ways. These BMNs present a new interest for nanomedicine, as they are being intensively studied. It was found that BMN are potent antioxidants and antimicrobial and anticancer agents [13].

Cancer chemoprevention implicates the use of pharmacological intervention to prevent, inhibit, or reverse carcinogenesis with synthetic or natural chemical products. Less than one percent of an estimated 250.000 plants has been screened pharmacologically. The use of phytochemicals present in medicinal plants as antioxidants possess no toxicity as compared to modern drugs and are efficient against different types of cancers with no side effects [14,15,16]. This study aims to bring together in vitro and in vivo results of the anticancer potential of BE and BMNs using BE.

## 2. Research Methodology

The literature data were collected via Clarivate Analytics, Science Direct, PubMed, and Google Academic (2011–2021). The search terms were: bark extracts, metallic nanoparticles, silver nanoparticles, gold nanoparticles, anticancer, cytotoxic activity, antiproliferative effect, and antimetastatic potential in vitro and in vivo. All of the search terms listed above were used in different combinations.

BioRender was used for drawing the figures from manuscripts (https://app.biorender.com/, accessed on 31 August 2021) [17].

## 3. Anticancer Potential of Bioactive Compounds from Bark Extracts (BE) in Different Type of Suppressed Cancer Cells Lines

The plant kingdom includes a multitude of secondary compounds such as polyphenols, alkaloids, flavonoids, lignans, terpenes, and taxanes. The list continues with minerals, vitamins, oils, and other biomolecules with great anticancer potential. The anticancer potential is due to potent antioxidant activity, inhibition of cancer cell-activating proteins, activating the repair DNA mechanism, or stimulating the formation of protective enzymes of biomolecules present in BE [18].

One of the important sources of bioactive compounds is forestry waste, especially bark [19,20]. Phenolic compounds as phenolic acids, flavonoids, lignans, and stilbenes, can capture free radicals and neutralize them and can prevent the aging process of cells and protect them from carcinogens [21]. There were more than 8000 phenolic compounds present in plants and their antioxidant effect has been observed in many studies [22]. The results suggested that an increased number of phenolic compounds from BE was correlated with their anticancer activity [23]. It is shown in the literature that a combination of two or three polyphenols has a synergistic and more efficacious effect in cancer treatment than using a single one as therapy [24]. One of the combinations used was EGCG (epigallocatechin-3-gallate) and quercetin [25,26] or ECGC and resveratrol against prostate cancer cells [27]. When using similar or complementary bio compounds the anticancer effect is enhanced; the anticancer effect is managed on multiple mechanisms of action [24]. Elansary et al. [28] studied expressly the polyphenolic characterization of *Malus baccata* and *Malus toringoides*. The three main polyphenols found in BE were protocatechuic acid, gallic acid, and catechin. They exerted potent anticancer activity against breast cancer, cervical cancer, and Jurkat T cell leukemia cancer cell lines, also having antioxidant and antimicrobial capability. The same authors found in another study that *Quercus* species showed an increased profile of polyphenol acids such as ellagic acid, gallic acid, protocatechuic acid, vanillic acid, and caffeic acid. These bio compounds showed necrosis and apoptotic effect in cancerous cells after treatment in a dose-dependent manner [29].

The alkaloids showed a beneficial effect on normal cells and cytotoxic activity against cancerous cells. This chemical family is one of the most important biologically active secondary metabolites found in the plant kingdom with multilateral biological activities [30,31]. Gabunamine, gabunine, and tabernamine from *Tabernaemontana johnstonii* BE exhibited important cytotoxicity against the P-388 mouse lymphocytic leukemia cell culture system [32]. Paclitaxel obtained from BE a few decades ago is part of this family of active anticancer bio compounds and was used in the treatment of ovarian cancer. Over time this isolate compound was approved to be used in other types of cancer [33].

Terpenes are another class of bio compounds in the alkaloid family with anticancer properties. Monoterpenes can act synergistically with polyphenols or alone with great potential in anticancer activity [34]. Over 20.000 terpenes were isolated and tested as anticarcinogens and the most important are lupeol, betulin, betulinic acid, oleanoic acid, etc. [35]. It was found that monoterpenes and sesquiterpenes found in Khaya senegalensis BE exhibited anticancer activity against colorectal and cervical cancer cell lines. Another study reported that terpenes have been introduced already in clinical testing for the treatment of solid tumors [36]. The BE biocompounds summarized in this review showed their potent activity as anticarcinogens. Some phytochemicals already are used in clinical therapy while others are used in clinical trials and some are barely discovered as potent anticarcinogens [37]. The BEs are found to be a valuable source of biologically active compounds with anticancer potential (Table 1).

Being the most pronounced cancer among women, breast cancer is one of the most studied cancer types. MCF-7 and MDA-MB-231 human breast cancer cell lines are often used in experiments. Table 1 shows the variety of BE with potential against breast cancer cell lines. For example, Ydav et al. [56] showed that *Saraca indica* L. (Fabaceae) alcoholic BE inhibited the proliferation of breast cancer cell lines. Its activity was more prominent in MCF-7 than in MDA-MB-231 cells.

Human lung carcinoma cell migration was inhibited in a dose-dependent manner by an ultrasound-assisted extract from *Fagus sylvatica* L. (Fagaceae) and *Picea abies* L. (Pinaceae) bark. In this study, the cytotoxic effect was not observed in the A549 lung carcinoma cell line but a slight modification was observed by increasing the concentration of the samples. In another study methanolic BE of *Costus pictus* D.Don (Costaceae) remarkably decreased cell viability in A549 lung carcinoma cell line in a dose-dependent manner [46].

PANC-1 was one of the pancreatic cancer cell lines inhibited by the β-caryophyllene compound. This was obtained by Dahham et al. [41] from essential oil from stem BE of *Aquilaria crassna*. The effect was similar to one of 5-fluorouracil, betulinic acid, and tamoxifen. Dahham et al. [41] showed that β-caryophyllene from the *Aquilaria crassna* bark had an antiproliferative effect mostly against HCT 116 and HT29 colon cancer cell lines. Because of the effect against colon cancer cell lines, they tested the efficacy of β-caryophyllene on the HCT 116 cell line. The recorded result was that β-caryophyllene can cease colonization with its cytotoxic effect.

## 4. Biosynthesized Metallic Nanoparticles (BMN) Mediated by Bark Extracts as Anticancer Agents

BEs are increasingly used in metallic nanoparticle biosynthesis as bioactive compounds that have the ability to reduce metallic ions. Because these biomolecules are ecofriendly and safe for human therapeutic use, the low cost makes it suitable for large-scale production over other bioactive products [13,62,63]. BMNs are used for their potential applications as antimicrobial, antioxidant, and anticancer agents [64].

More than 65% of nanoparticles used in general biological applications are silver NPs as earlier studies have shown. That is because they have the smallest dimension and have easy access to permeate the cellular membrane and affect cellular physiology. When the diameter of a nanoparticle decreases the touching surface increases, and that has an explicit influence on their cell entrance and efficacy [63]. Beyond their nanosize, the BMN has another advantage. The biomolecules present in BE are potent stabilizing agents that are attached to the surface of the nanoparticles and also have a cytotoxic effect. That effect is combined with the size advantage and the cytotoxic effect is highlighted [65].

Silver nanoparticles have an important role in therapy for wound healing, not only in skin but also in breast cancer. In this review, the majority of cited studies are on silver NPs and the principally tested cells are breast cancer cell lines. For example, the BE AgNPs from *Elaeodendron croceum* displayed an IC50 value of 138.8 µg/mL against the MDA-MB-231 breast cancer cell line, comparable with the IC50 value of 80 µg/dL obtained after treatment with Paclitaxel [66].

Another study showed that approximately 82.5% of A549 lung cancer cells were dead after treatment with *Toxicodendron vernicifluum* (Stokes) F.A. Barkley (Anacardiaceae) AgNPs [67]. Table 2 summarizes the potential anticancer activity of BMN, specifying the type of suppressed cancer cells.

## 5. BE and BMNs Mechanism of AntiCancer Action

**Figure 1:**
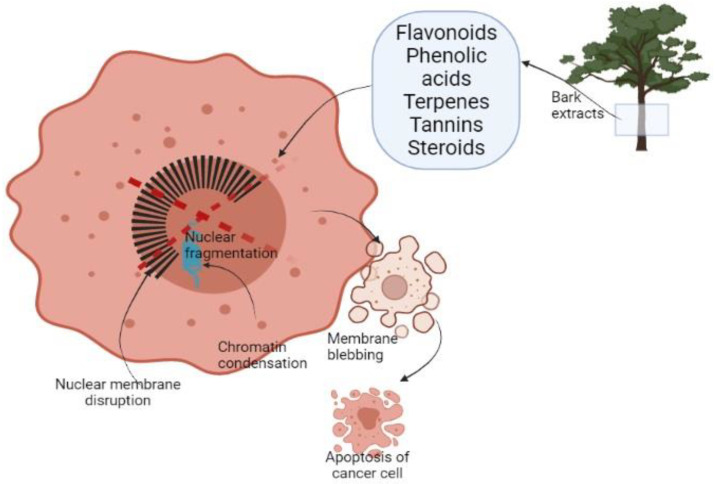
Schematic representation of cancer cell apoptosis after treatment with BE.

It has been reported that plant-derived extracts show a selective decrease in the growth activity of cancer cells compared to normal cells [38]. This selective targeting is due to phytochemicals present in plants as flavonoids, phenolic acids, terpenes, tannins, and steroids (Figure 1). These compounds are described as key antioxidant agents. The reasons for selective annihilation of cancerous cells and nontoxicity on normal ones remain yet unknown [46,80]. The inducing antioxidant activity of natural extracts mainly described for redox activity acts as a reducing agent, hydrogen donor, or oxygen scavenger. That might be helpful in preventing or slowing down the evolution of oxidative stress-related disorders as cancer [46,59].

Cell death appears by two alternate modes apoptosis or necrosis. These two ways are different because the first one is programmed managed cell death without a local inflammatory response. Necrosis is characterized as a passive unexpected form of cell death dependent on environmental disturbance with the unrestrained release of inflammatory response. Apoptotic cells go through diverse morphological transformations such as membrane blebbing, chromatin condensation, nuclear membrane disruption, nuclear fragmentation, and dissolution (Figure 1). The first sign of apoptosis is the apoptotic body [81]. Cell apoptosis develops through the mitochondria-mediated intrinsic pathway or death receptor-induced extrinsic pathway. It was observed that BE from *Pinus massoniana* has a role in both pathways. After the treatment of HeLa cells the expression of Bax, a proapoptotic protein vigorously increased. Cell apoptosis was induced by releasing of cytochrome C [82,83].

The molecular mechanism of BE from *Spondias pinnata* mediated apoptosis induction was observed in A549 lung carcinoma and MCF-7 breast cancer cell lines. The western blot results showed an increased expression of Bax (proapoptotic) protein and a decreased one for Bcl-2 (antiapoptotic) protein in A549 cells. The same expression was observed after treatment in MCF-7 cells. The only difference was in Bcl-2 expression that was not changed [58]. Alvala et al. [60] found that the BE of *Tecomella undulata* caused apoptosis on the K562 chronic myeloid leukemia cell line. That was evident after DNA fragmentation assay, increase in FAS (cell surface death receptor), FADD (FAS associated death domain protein) levels, and activation of caspase 8 and 3/7 (a cysteine protease that initiates apoptotic signaling via the extrinsic apoptotic pathway).

*Alstoria scholaris* and *Alstoria venenata* BE showed important membrane blebbing, nuclear condensation, and damage, which are manifestations of apoptosis rather than necrosis. The cytotoxic effect of BE on DLA (Dalton`s ascitic lymphoma) cells and their cytoprotective effect on normal ones may be due to their potent antioxidant activity [39].

**Figure 2:**
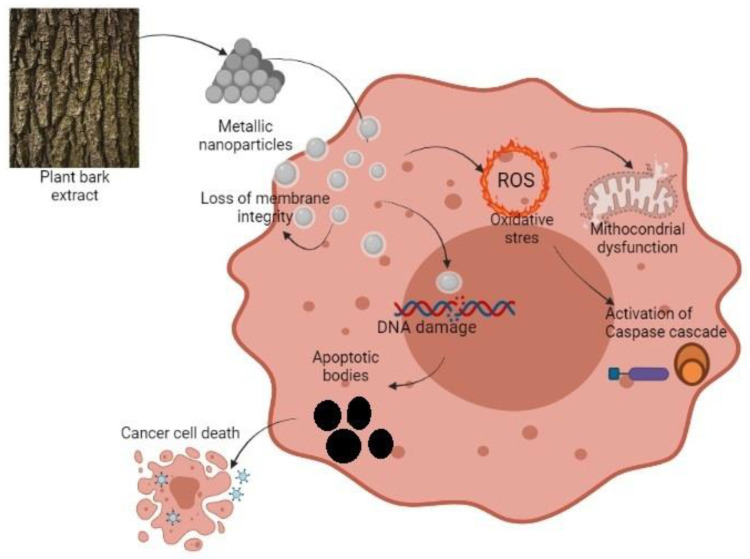
Schematic representation of BMN action against a cancer cell.

Nayak et al. [65] studied the mechanism of silver nanoparticles obtained from the bark extracts of *Azadirachta indica* on the MG-63 osteosarcoma cell line after 24 h of treatment. They observed the formation of granulations in the nucleus, which is a measure of chromatin condensation. That reaction is well documented in nanoparticle treated samples before. The cytotoxic activity of metallic nanoparticles is due to their small size and affinity for the acidic pH of the cancer cells [84]. It is known that a characteristic of apoptosis is the loss of mitochondria membrane integrity and potential (Figure 2). The fluorescence signal decreases when the mitochondria lose their membrane potential and the absorption of rhodamine 123 by the affected cells is low. β-caryophyllene from *Aquilaria crassna* essential oil BE had a potent capacity in apoptosis-inducing. The fluorescence signal decreased in treated cancer cells PANC-1 pancreatic cancer, HCT-116, and HT29 colorectal cancer. The untreated cells showed a substantial fluorescence intensity. These results present the unaffected state of normal cells [40]. In the same way, another study revealed that β-caryophyllene oxide produces apoptosis. They found that apoptosis is produced through suppression of PI3K/AKT/mTOR/S6K1 pathways and also following ROS-mediated MAPKs activation. The first signaling pathway is not only conducting toward apoptosis but is closely related to angiogenesis and the second pathway ROS-mediated mitogen-activated protein kinases activation regulates diverse cellular actions as proliferation, motility, and survival [85].

In another study [86], the effect of BE from *Acanthopanax sessiliflorus* on MDA-MB-231 and MCF-7 breast cancer cell lines was shown. The intracellular ROS (reactive oxygen species) levels were determined by inspecting the fluorescent intensity of a redox reagent. It was observed that the BE elevated the proportion of green fluorescence in both cell lines dose-dependently. That suggests that the studied BE stimulates the production of intracellular ROS after the selective interruption of the mitochondrial respiratory chain. This is evidence of apoptosis with the involvement of mitochondrial membrane depolarization. In general, ROS contributed to cell death inducing the cell membrane lipid oxidation and the expression of genes associated with DNA disruption, which promotes DNA damage in apoptosis or necrosis at levels above the cellular antioxidant defense systems [8,86].

Another way for cancer cell extinction is interrupting the cell cycle. After the analysis of cellular cycles, BE from *Euphorbia umbellata*, showed that chloroform fraction promotes cell cycle arrest at G0/G1 phase. Moreover, treatment with this potent extract showed a decreased percentage of cells in S-phase [48]. Another study conducted by Gonzalez-Sarrias et al. [38] revealed that proliferation of colon cancer cells can be inhibited by the compounds present in the maple BE, by arresting the advancement of the cell cycle at the S-phase. In addition, cancer cells own an extra survival mechanism such as colonization, migration, tumor angiogenesis, cell adhesion, epithelial-mesenchymal transition, and transendothelial migration known as a metastatic cascade. The suppression of colonization and migration of HCT 116 colon cancer cells was demonstrated by β-caryophyllene. It also had the ability to prevent metastatic propagation of malignant cells and to suspend cell invasion, the main characteristic of metastatic cascade [41,87,88].

Toxicity on different normal cells was realized to observe the behavior of the BE. For example, cytotoxicity of the *Toxicodendron vernicifluum* BE was studied. The results indicated that NIH3T3 mouse embryonic fibroblasts growth was not significantly reduced and cell death was not observed at different concentrations of the extract [67].

The β-caryophyllene found in essential oil from *Aquilaria crassna* bark exhibited low toxicity against 3T3-L1 and RGC-5 normal cell lines. The results showed that even the higher concentration of β-caryophyllene did not affect the morphology of a normal cell line (3T3-L1) [41]. In the case of the HaCaT human keratinocytes normal cell line, the BE from *Fagus sylvatica* and *Picea abies* did not show cytotoxic activity. Instead, BE stimulated normal cells’ proliferation [49].

The in vivo toxicity of AgNPs bark extract of *Elaedendron croceum* was assessed on male Wister rats. The authors revealed that there was no mortality or any toxic reaction after the acute oral test. There was no toxicity observed in the heart, liver, and kidney after the morphological investigation [66]. Another efficacy advantage is that during chemical reactions in the synthesis of nanoparticles, some harmful reactants from BE are involved in capping and reducing the AgNPs. Therefore, the cytotoxic effect of AgNPs on normal cells is reduced [66]. In addition, it was observed in the literature that NPs with extremely small diameters appear to have a higher cytotoxic effect in normal cells due to swift internalization than NPs with higher diameter [89].

Accordingly, with data shown in studies, the tests effectuated on normal cells with BE and BEM exhibited no harmful or toxic effects.

## 6. Conclusions and Future Perspectives

Cancer has become a front line between resistance to chemotherapy, aggressive invasion, secondary toxic effects, and scientists who struggle to find a better chemical bioactive extract to maintain homeostasis, to avoid the unwanted secondary toxic effects, and to prevent metastatic cascade. BEs and BMNs are a future promise source of antioxidant compounds, which can fight against cancerous cells. All standardized extracts, shown in the listed studies above, had an impressive antioxidant capacity that was linked with anticancer ability. For maximal certainty, researchers tested in vitro the BEs and BMNs cytotoxic effect on cancer cells and normal cells. Unfortunately, in vivo studies for the anticancer potential of BEs are few. The results are encouraging and in vivo experiments should be supported. BMNs showed also antioxidant potency with great in vitro cytotoxic effect on cancer cells but no in vivo experiment was found. The toxicity on normal cells was shown as harmless. This is a promising result for the application of BEs and BMNs on animal subjects and bypasses the secondary undesirable effect of chemotherapy. There remains a need for in vivo studies to confirm the anticancer action of BEs and BMNs.

## Figures and Tables

**Table 1:** Type of cancer cells lines suppressed by bioactive compounds from BE.

Source of Bark: Scientific Name (Family)—Common Name	Extracts Type	Main Bioactive Compounds	Experiment Type	Type of Suppressed Cancer Cells Lines	References
*Acanthopanax sessiliflorus* (Decne & Planch) Miq. (*Araliaceae*)—thorny ginseng	n-hexane fraction of stem bark methanolic extract	-	In vitro	MDA-MB-231 and MCF-7 human breast cancer	[8]
*Acer rubrum* L. *(Sapindaceae)*—red maple	Methanolic extract	Polyphenols—ginnalin-A	In vitro	HCT-116 Caco-2 HT-29 colon cancer cell lines	[38]
*Acer saccharum* March. *(Sapindaceae)*—sugar maple	Methanolic extract	Polyphenols—ginnalin-A	In vitro	HCT-116 Caco-2 HT-29 colon cancer cell lines	[38]
*Alstonia scholaris* (L.) R.Br. (*Apocynaceae*)—devil`s tree	Hexane extract		In vitro	DLA: dalton`s lymphoma ascitic cell line	[39]
*Alstonia venenata* R. Br. (*Apocynaceae*)	Hexane extract		In vitro	DLA: dalton`s lymphoma ascitic cell line	[39]
*Antocephalus cadamba* (Roxb.) Miq. (*Rubiaceae*)—kadam	Petroleum ether Methanolic extract	-	In vivo on Swiss albino mice	EAC: Ehrlich ascites carcinoma	[40]
*Aquilaria crassna* Pierre *(Thymelaeaceae)*—Agar wood	essential oil from stem bark extract	β-caryophyllene	In vitro	PANC-1 pancreatic cancer, HCT-116 and HT29 colorectal cancer	[41]
*Balanite aegyptiaca* (L.) Delile *(Balamitaceae)*—desert date	Choloform Methanolic water Hexane extract	Alkaloids	In vivo antitumor activity in nude mice induced with HCT-116 tumor	HCT-116 colon cancer K562 myelogenous leukemia U937 histyocitic lymphoma MCF-7 breast cancer	[42]
*Betula pendula* Roth *(Betulaceae)*—common silver birch	2-propanol and ethanolic extract	Betulin and betulinic acid	In vitro	HeLa human cervical cancer; A431 human squamous carcinoma; A2780 ovarian carcinoma; MCF-7 breast cancer	[37]
*Betula utilis* D.Don *(Betulaceae)*—Himalayan silver birch	Ethyl acetate extract	Triterpenes	In vitro	MCF-7	[6]
*Canarium odontophyllum* Miq. (*Burseraceae*)—dabai	Acetone extract	Flavonoids Saponins Tannins Terpenoids and phenolic compounds	In vitro	HCT 116 colorectal cancer	[43]
*Catalpa speciosa* Warder *(Bignoniaceae)*—northern catalpa	Methanolic bark extract	Phenolic acids catechins	In vitro	HeLa cervical cancer; MCF-7 breast cancer; Jurkat leukemic T cells; T24 urinary bladder carcinoma; HT-29 colorectal adenocarcinoma	[44]
*Cinnamomum zeylanicum* Blume. *(Lauraceae)*—true cinnamon tree	Methanolic extract	-	In vitro	HepG2 hepato carcinoma	[45]
*Costus pictus* D.Don *(Costaceae)*—painted spiral ginger	Methanolic extract	Polyphenols Flavonoids	In vitro	HT-29 colon cancer; A549 lung carcinoma	[46]
*Crysophillum perpulchrum* L. (*Sapotaceae*)	Methanolic bark extract	Polyphenols	In vitro	HeLa cervical cancer	[47]
*Euphorbia umbellata* (Pax) Bruyns *(Euphorbiaceae)*—African milk bush	Water and methanolic extract	Triterpenes Steroids	In vitro	Jurkat cells T-cell leukemia	[48]
*Fagus sylvatica* L. *(Fagaceae)*—beech	Water bath extraction and ultrasound-assisted extraction	Polyphenols	In vitro	A375 melanoma; A549 lung carcinoma	[49]
*Ficus drupacea* Thunb. *(Moraceae)* —brown-woolly fig	Methanolic extract, n-hexane fraction	Oleanolic acid, friedelin, and epilupeol acetate	In vitro	HeLa cervical cancer; MCF-7 breast cancer; Jurkat leukemic T cells; HT-29 colorectal cancer; and T24 urinary bladder carcinoma	[50]
*Khaya senegalensis* (Desr.) A Juss *(Meliaceae)*—African mahogany	Methanolic, water, and SFE (subcritical fluid extraction)	Monoterpenes Sesquiterpene	In vitro	Caco 2 colorectal cancer; HeLa cervical cancer	[51]
*Magnolia acuminata* L. *(Magnoliaceae)*—cucumber tree	-	Phenolic acids—protocatechuic acid Catechin Epicatechin	In vitro	HeLa cervical cancer; MCF-7 breast cancer; Jurkat leukemic T cells; T24 urinary bladder carcinoma; HT-29 colorectal adenocarcinoma	[44]
*Malus baccata var.gracilis* (Rehder) T.C.Ku. (*Rosaceae*)—Siberian crab apple	Methanolic extract	Polyphenols protocatechuic acid, gallic acid, and catechin	In vitro	MCF-7 breast cancer cells, HeLa cervical cancer Jurkat cells T cell leukemia	[28]
*Malus toringoides* Hughes (*Rosaceae*)—cut-leaf crabapple	Methanolic extract	Polyphenols protocatechuic acid, gallic acid, and catechin	In vitro	MCF-7 breast cancer cells, HeLa cervical cancer Jurkat cells T cell leukemia	[28]
*Mangifera zeylanica* (Blume) Hook. f (*Anacardiaceae*)— Sri Lanka wild mango	Hexane, chloroform, ethyl acetate and methanol extract	Steroids, flavonoids, phenolic compounds, and tannins	In vitro	MCF-7 and MDA-MB-231 breast cancer SKOV-3 ovarian cancer	[1]
*Moringa oleifera* Lam. *(Moringaceae)*—drumstick tree	Hexane and benzene extract	-	In vitro	DLA: dalton`s lymphoma ascitic cell line	[39]
*Moringa oleifera* Lam. *(Moringaceae)*—drumstick tree	Ethanolic extract	Eugenol, isopropyl isothiocynate, D-allose, and hexadeconoic acid ethyl ester	In vitro	MDA-MB-231 breast cancer HCT–8 ileocecal adenocarcinoma	[52]
*Margaritaria* discoidea (Baill.) G. L. Webster *(Euphorbiaceae)*—pheasant-berry	Dichloroethane and methanol extract	Gallic acid Securinine	In vitro	OVCAR-8; A2780 ovarian cancer cell lines A2780cis cisplatin resistant ovarian cancer	[53]
*Oroxylum indicum* (L.)Benth. Ex Kurtz *(Bignoniaceae)*—Indian trumpet flower	Petroleum ether Dichloromethane Methanol extract	Polyphenols Flavonoids	In vitro	HeLa cervical adenocarcinoma	[54]
*Picea abies* L. *(Pinaceae)*—spruce	Water bath extraction and ultrasound-assisted extraction	Polyphenols, tannins, flavonoids, and flavonols	In vitro	A375 human melanoma; A549 lung carcinoma	[49]
*Pinus massoniana* Lamb. *(Pinaceae)*—chinese red pine		Flavonoids (Tannins)—proanthocyanidins	In vitro	HeLa cells cervical cancer	[55]
*Quercus acutissima* Carruth. (*Fagaceae*)—Sawtooth oak	Methanolic extract	Phenolic acid—scaffeic acid, ellagic acid, gallic acid, and protocatechuic acid	In vitro	MCF-7 breast cancer; HeLa Cervical cancer Jurkat T-cell leukemia cell line	[29]
*Quercus macrocarpa* Michx. *(Fagaceae)*—bur oak	Methanolic extract	Phenolic acids—caffeic acid and catechins	In vitro	MCF-7 breast cancer; HeLa Cervical cancer Jurkat T-cell leukemia cell line	[29]
*Quercus robur* L. *(Fagaceae)*—common oak	Methanolic extract	Phenolic acids—ellagic acid, gallic acid, protocatechuic acid, and vanillic acid	In vitro	MCF-7 breast cancer; HeLa Cervical cancer Jurkat T-cell leukemia cell line T24 bladder transitional cell carcinoma	[29]
*Saraca indica* L. *(Fabaceae)*—asoka-tree	Alcoholic extract	Polyphenols	In vitro	MCF-7, MDA-MB-231 breast cancer	[56]
*Sesbania grandiflora* (L.) Poiret *(Fabaceae*)—vegetable hummingbird	Ethyl alcoholic extract	Phenolics terpenoids and phenolics flavonoids	In vitro	MCF-7 human breast cancer and HL-60 human leukemia	[57]
*Spondias pinnata* L.f.Kurz *(Anacardiaceae)*—wild mango	Water methanolic extract	-	In vitro	A549 lung carcinoma; MCF-7 breast carcinoma	[58]
*Stryphnodendron adstringens* (Mart.) Coville *(Fabaceae)*—barbatimao	Water extract	Proanthocyanidins (Tannins) Triterpenoids Gallic acid Gallocatechin epigallocathechin	In vitro	B16F10Nex-2 melanoma cells	[59]
*Taxus cuspidata* Siebold &Zucc *(Taxaceae)*—Japanese yew		Phenolic acids	In vitro	HeLa cervical cancer; MCF-7 breast cancer; Jurkat leukemic T cells; T24 urinary bladder carcinoma; HT-29 colorectal adenocarcinoma	[44]
*Tecomella undulata* (Sm.) Seem. *(Bignoniaceae)*—desert teak	Chloroform extract	Steroids and Triterpenes Flavonoids	In vitro	K562 erythroleukemic cell line	[60]
*Theobroma cacao* L. (*Malvaceae*)—cocoa tree	Methanolic extract	Flavonoids Saponins Triterpenes Condensed tannins Steroids	In vitro	MCF-7 breast cancer	[61]
*Wrghtia tinctoria* (Roxb.)R. Br. *(Apocynaceae)*—pala indigo tree	Ethyl alcoholic extract	Alkaloids terpenoids Phenolics	In vitro	MCF-7 human breast cancer	[57]

**Table 2:** Type of cancer cell lines suppressed by biosynthesized metalic nanoparticles (BMN) mediated by bark extracts.

Source of Bark: Scientific Name (Family)—Common Name	BMNs Type	Experiment Type	Type of Suppressed Cancer Cell Lines	References
*Albizia chevaalieri* Harms (*Fabaceae*)	Ag	In vitro	MDA-MB231, MCF-7 breast cancer and HepG2 liver cancer	[68]
*Albizia lebbeck* L. Benth. (*Fabaceae*)—lebbeck tree	ZnO	In vitro	MDA-MB-231; MCF-7 breast cancer	[69]
*Azadirachta indica* A.Juss. *(Meliaceae)*—Nimtree or Indian lillac	Ag	In vitro	MG-63 osteosarcoma	[65]
*Elaeodendrum croceum (Thunb.) DC. (Celastraceae)* —saffron	Ag	In vitro	MDA-MB-231 breast cancer	[66]
*Ficus benghalensis L. (Moraceae)*—banyan fig	Ag	In vitro	MG-63 osteosarcoma	[65]
*Ficus benghalensis var. krishnae* L. (*Moraceae*)—Krishna`s butter cup	Ag	In vitro	SKOV-3 ovarian cancer	[70]
*Garcinia mangostana* L. (*Clusiaceae*)—Mangosteen	Ag	In vitro	A549 lung cancer	[71]
*Moringa oleifera* L. *(Moringaceae)*—drumstick tree	Ag	In vitro	HeLa cervical cancer	[72]
*Nerium oleander* L. *(Apocynaceae)*—Karabi	Au	In vitro	MCF-7 breast cancer	[73]
*Prosopis juliflora* Sw. DC. (Fabaceae)—Mesquite	Ag	In vitro	A549 lung cancer	[74]
*Stereospermum suaveolens* Roxb. DC.—(*Bignoniaceae*)	Ag Au	In vitro	A549 lung cancer	[75]
*Syzygium alternifolium* (Wt.) Walp *(Myrtiaceae)*—North Arcot	Cu	In vitro	MDA-MB-231 breast cancer	[76]
*Terminalia arjuna* Wigh and Arn (*Combretaceae*)—Arjuna tree	Cu–Ag	In vitro	MDA-MB-231 breast cancer; HeLA cervical cancer; SiHa squamous cell carcinoma; HepG2 liver carcinoma	[77]
*Terminalia mantaly* H. Perrier *(Combretaceae)*—Madagascar almond, umbrella tree	Au	In vitro	Caco-2 colon cancer; MCF-7 breast cancer; HepG2 liver cancer	[78]
*Toxicodendron vernicifluum* (Stokes) F.A. Barkley (Anacardiaceae)—Chinese lacquer tree	Ag	In vitro	A549 Adenocarcinomic human alveolar basal epithelial cells	[67]
*Salacia chinensis* L. *(Celastraceae)*—Chinese salacia	Ag	In vitro	HepG2 liver cancer; L-132 lung cancer; MIA-Pa-Ca-2 pancreas cancer; MDA-MB-231 breast cancer; KB cells oral cancer; PC-3 prostate cancer; HeLa cervical cancer cells	[79]

Ag: silver nanoparticles; Au: gold nanoparticles; Cu: copper nanoparticles; Cu–Ag: copper and silver bimetallic nanoparticles; ZnO: zinc oxide nanoparticles.
